# The relationship between nicotine dependence and willingness to quit smoking: A cross-sectional study

**DOI:** 10.1016/j.pmedr.2025.103066

**Published:** 2025-04-06

**Authors:** Afraa Murriky, Eman Allam, Hind Alotaibi, Razan Alnamasy, Atheel Alnufiee, Amjad AlAmro, Amal Al-hammadi

**Affiliations:** a Department of Restorative Dentistry, Riyadh Elm University, Riyadh, Kingdom of Saudi Arabia; b Research and Graduate Studies Department, Mohammed Bin Rashid University of Medicine and Health Sciences, Dubai, United Arab Emirates

**Keywords:** Nicotine dependence, Smoking cessation, Willingness to quit, Adult Saudi population

## Abstract

**Objective:**

To assess the relationship between nicotine dependence and willingness to quit smoking in a sample of adult Saudi population and identify the primary motivations and barriers affecting their decision and willingness to quit smoking.

**Methods:**

Data collection was conducted from March 1 to March 30, 2023, through an online questionnaire designed to predict the relationship between nicotine dependence and willingness to quit smoking. Participants included adults who smoke (≥ 18 years of age, *n* = 289). The questionnaire included three sections covering basic demographic information; the willingness to quit in the next 30 days with reasons; and items related to the Fagerstrom test for nicotine dependence. A multiple logistic regression model estimated the adjusted effects of sex, age, education, duration of smoking, and level of nicotine dependence on the willingness to quit.

**Results:**

A high level of nicotine dependence was recorded for 38 participants (13 %). No significant differences were reported in the participants' characteristics between those who had the willingness to quit smoking in the next 30 days and those who had not. Health issues was the most selected reason for willingness to quit smoking (75.2 %). Social and psychological factors such as enjoying smoking (74.2 %), friends are smoking (21.9 %), followed by depression (17.2 %) were among the reported reasons for not being willing to quit smoking.

**Conclusions:**

While demographic characteristics, duration of smoking, and nicotine dependence level did not significantly correlate with participants' willingness to quit, health concerns was a key motivator. Significant barriers to the smoking cessation decisions appeared to be related to social influences and psychological challenges.

## Highlights

• No link between demographics, start date, dependence, and willingness to quit.
• Personal health concerns drove motivation to quit smoking.
• Social and psychological factors were key barriers to quitting.

## 1 Introduction

Smoking and tobacco use is a public health concern and one of the most significant epidemics worldwide. It is linked to numerous preventable diseases and is said to be responsible on killing more than eight million people globally on annual basis. Direct tobacco uses accounts for about seven million of the deaths, whereas second-hand smoke exposure for people who do not smoke accounts for another 1.3 million (Siddiqi et al., 2020; National Center for Chronic Disease Prevention and Health Promotion, 2019). From healthcare point of view, there is no level of tobacco exposure that is safe, and all kinds of tobacco use are considered hazardous.

The most prevalent type of tobacco usage is cigarette smoking. Waterpipe tobacco, cigars, cigarillos, heated tobacco, roll-your-own tobacco, pipe tobacco, and smokeless tobacco products are other forms of tobacco use. In addition to the well-documented adverse health effects, attributable economic costs of tobacco use are devastating. These range between direct medical expenses to costs associated with loss of productivity. Around 80 % of tobacco users worldwide live in low- and middle-income countries (Siddiqi et al., 2020; Banks et al., 2015).

Tobacco dependence is a chronic addictive condition. After quitting smoking for an extended period, people who smoke will experience several withdrawal symptoms, including irritability, insomnia, mood swings, and inability to concentrate, which makes it difficult for them to stop in most cases (Li and Wang, 2020). People with nicotine dependence are likely to experience urges or cravings in stressful situations.

The extent of nicotine dependence is one of the main factors determining whether a smoker can successfully quit smoking (Ma et al., 2020). According to the score of a validated tool such as the Fagerstrom test for nicotine dependence.

(FTND), nicotine dependence level can be estimated, and management can be recommended. People who smoke with low-level nicotine dependence frequently receive cognitive behavioural therapy (CBT). Moderate to heavy nicotine dependence require CBT together with a pharmacotherapeutic agent and people who smoke unwilling to quit nicotine are subject to motivational interviews (Mushtaq and Beebe, 2017).

Reports indicated that, the frequency and quantity of smoking increase with the severity of nicotine dependence (Li et al., 2020; Ma et al., 2020). A study demonstrated that after two to three years of quitting smoking, the risk of developing stroke decreased and continued to decline more significantly after quitting smoking for more than five years (Xu et al., 2018). An essential first step to changing individuals' behaviour toward smoking is their willingness to stop. It is well recognized that willingness to quit is strongly linked to future attempts to quit and ultimately cease smoking (Li et al., 2020; Ma et al., 2020). The aim of this study was to assess the relationship between nicotine dependence and willingness to quit smoking in a sample of adult Saudi population and identify the primary motivations and barriers affecting their decision and willingness to quit smoking. While there have been many studies of motivations and barriers associated with willingness or intention to quit in United States adults and other Western populations, this study extends the knowledge about smoking in Saudi adults.

## 2 Methods

This study employed a cross-sectional methodology utilising a survey designed to predict the relationship between nicotine dependence and willingness to quit smoking. Data collection was conducted from March 1 to March 30, 2023, through an online questionnaire distributed to adults who smoke (≥ 18 years of age). The questionnaire was distributed among adults reporting to the outpatient clinic of Riyadh Elm University (REU), and data were collected prospectively and anonymously based on informed consent and voluntary participation. The project was approved by the institutional review board committee of REU, Riyadh, Kingdom of Saudi Arabia (KSA).

The questionnaire included three sections covering basic demographic information; the willingness/ unwillingness to quit in the next 30 days with reasons; as well as items related to the Fagerstrom test for nicotine dependence (FTND). Data collected included participants' age, sex, educational level, duration of smoking (years), and drivers of the willingness/unwillingness to quit smoking. A dichotomous question (yes/no response) was utilized to investigate the respondents' intention to consider quitting in the next 30 days.

The level of nicotine dependence was assessed using a reliable and validated tool which is the FTND (Heatherton et al., 1991; Etter, 2005; Fagerstrom et al., 2012). The test includes a total of six items, and the score varies between 0 and 10 where: a 0 to 3 score indicates mild nicotine dependence, 4 to 6 score indicates moderate nicotine dependence, and a 7 to 10 score represents severe nicotine dependence and more serious smoking related adverse consequences.

### 2.1 Statistical analysis

The reliability of the assessment tool was evaluated using test-retest approach where trained and calibrated investigators collected responses from ten cases at two different occasions with two weeks in-between. The process was repeated until an intraclass correlation coefficient value of 0.8 was reached for all questions. Descriptive statistics of the total sample were performed, and categorical variables were presented as counts and percentages. The chi-square test was used to determine associations between the willingness to quit and each of the following: sex, age, education, the duration of smoking (years), and level of nicotine dependence. A multiple logistic regression model was conducted to estimate the adjusted effects of sex, age, education, duration of smoking (years), and level of nicotine dependence on the willingness to quit; those were presented as adjusted odds ratios (AOR) with their 95 % confidence intervals (CI). In all analyses, a significance level of 0.05 was used. Data were analysed using the R software version 4.2.2 (The R Project for Statistical Computing, Vienna, Austria).

## 3 Results

The present study included 291 participants of which two were excluded from the analysis due to missing the response to the question regarding their willingness to quit smoking in the next 30 days. A total of 600 individuals were originally invited to participate in the study, of whom 291 agreed to take part, resulting in a participation rate of 48.5 %. The level of nicotine dependence was missing in 29 participants and was imputed using a random forest algorithm that included the sex, age, education, and duration of smoking (years), as predictors. Most of the participants were males (84 %) below 35 years of age (73 %). Only 8 % had at least a bachelor's degree. Most of the participants had been smoking for five years at least (79 %). A high level of nicotine dependence was recorded for 38 participants (13 %). No significant differences were reported in the participants' characteristics between those who had the willingness to quit smoking in the next 30 days and those who had not (Table 1).

**Table 1:** Characteristics of Saudi adults, stratified by willingness to quit smoking in the next 30 days (March 1 to March 30, 2023).

Variables	Total sample (*N* = 289)	Willing to quit (*N* = 161)	Not willing to quit (*N* = 128)	P
	n(%)	n(%)	n(%)	
Sex (male)	242(83.7)	137(85.1)	105(82.0)	0.59

Age category				0.92
18–24 years	92(31.8)	49(30.4)	43(33.6)	
25–34 years	118(40.8)	67(41.6)	51(39.8)	
35–44 years	57(19.7)	34(21.1)	23(18.0)	
45–54 years	12(4.2)	6(3.7)	6(4.7)	
≥ 55 years	10(3.5)	5(3.1)	5(3.9)	

Education				0.72
Intermediate	189(65.4)	103(64.0)	86(67.2)	
High school	78(27.0)	47(29.2)	31(24.2)	
Bachelor	7(2.4)	4(2.5)	3(2.3)	
Master	15(5.2)	7(4.3)	8(6.3)	

Started smoking				0.57
< 5 years	62(21.5)	32(19.9)	30(23.4)	
5 years	32(11.1)	18(11.2)	14(10.9)	
6–10 years	71(24.6)	42(26.1)	29(22.7)	
11–15 years	61(21.1)	38(23.6)	23(18.0)	
20 years	63(21.8)	31(19.3)	32(25.0)	

Nicotine dependence				0.96
High	38(13.1)	22(13.7)	16(12.5)	
Moderate	78(27.0)	43(26.7)	35(27.3)	
Low-moderate	73(25.3)	42(26.1)	31(24.2)	
Low	100(34.6)	54(33.5)	46(35.9)	

*P*-values were calculated using the chi-square test. Nicotine dependence was measured using the Fagerstrom test for nicotine dependence (FTND). “Intermediate” education refers to education completed up to the middle school.

To indicate predictors of the willingness to quit smoking, multiple logistic regression analysis was performed. The analysis estimated the adjusted effects of sex, age, education, the duration of smoking, and level of nicotine dependence on the likelihood of willingness to quit (Table 2).

**Table 2:** Predictors of the willingness to quit smoking in Saudi adults (March 1 to March 30, 2023).

Explanatory variables	AOR (95 % CI)	P
Nicotine dependence		
Moderate vs high	0.97 (0.41, 2.29)	0.95
Low-moderate vs high	1.03 (0.45, 2.37)	0.94
Low vs high	0.79 (0.35, 1.76)	0.57
Sex		
Male vs female	1.17 (0.60, 2.28)	0.65
Age		
25–34 vs 18–24 years	1.16 (0.60, 2.24)	0.66
35–44 vs 18–24 years	1.45 (0.62, 3.44)	0.39
45–54 vs 18–24 years	1.10 (0.27, 4.53)	0.89
≥ 55 vs 18–24 years	0.97 (0.23, 4.18)	0.97
Education		
High school vs intermediate	1.34 (0.76, 2.36)	0.31
Bachelor vs intermediate	1.37 (0.27, 7.66)	0.70
Master vs intermediate	0.77 (0.25, 2.35)	0.68
Smoking start		
5 vs < 5 years	1.23 (0.51, 2.99)	0.65
6–10 vs < 5 years	1.25 (0.57, 2.75)	0.59
11–15 vs < 5 years	1.38 (0.58, 3.30)	0.47
20 vs < 5 years	0.77 (0.32, 1.86)	0.56

CI: confidence interval; AOR: adjusted odd ratio; vs: versus. Nicotine dependence was measured using the Fagerström test for nicotine dependence (FTND). “Intermediate” education refers to education completed up to the middle school.

**Fig. 1:**
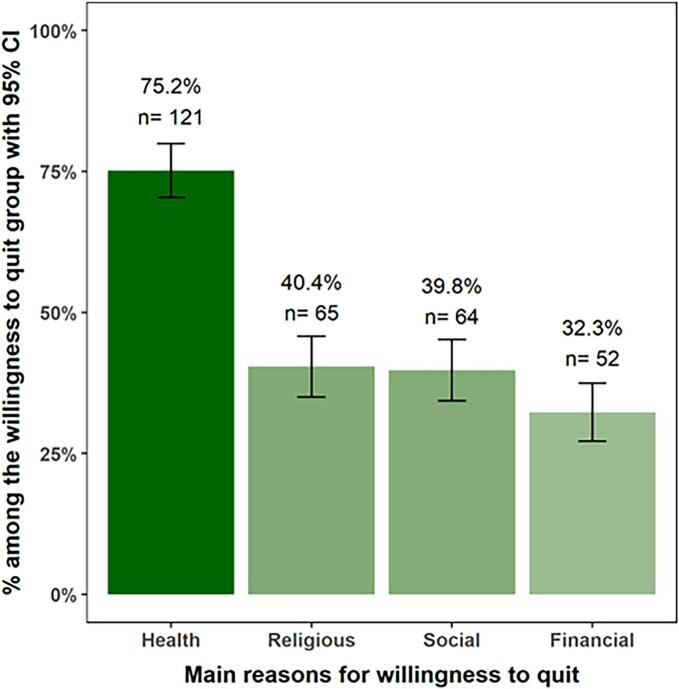
Factors associated with willingness to quit smoking in Saudi adults (March 1 to March 30, 2023).

**Fig. 2:**
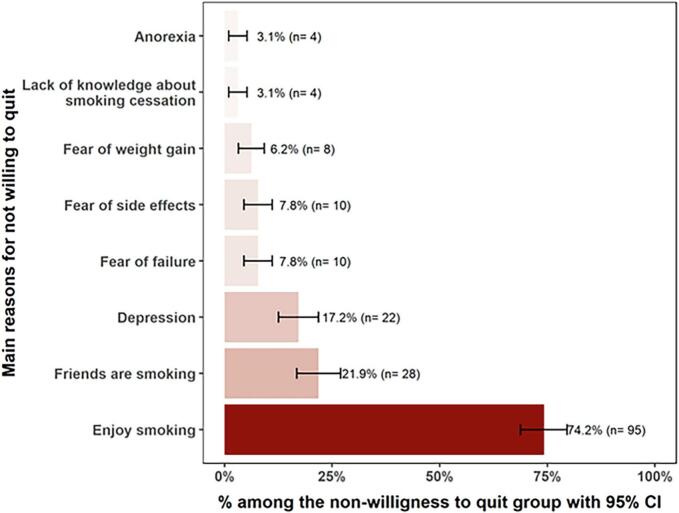
Factors associated with lack of willingness to quit smoking in Saudi adults (March 1 to March 30, 2023).

Fig. 1 shows the main reasons for the willingness to quit smoking. Health issues was the most selected reason (75.2 %). Fig. 2 demonstrates the reported reasons for not being willing to quit smoking which were mostly enjoying smoking (74.2 %), friends are smoking (21.9 %), followed by depression (17.2 %).

## 4 Discussion

The willingness to quit smoking among adults who smoke is influenced by a complex interplay of factors, including smoking habits in terms of frequency and intensity. An individual's biological, socioeconomic, and psychological characteristics also influence their decision to stop smoking. Smoking habits are usually part of the daily routines of people who smoke which makes cessation seems very challenging especially for those with well-established habits (Curry et al., 1990; Lucchiari et al., 2020; Huo et al., 2023). Understanding these diverse influences is crucial for developing targeted interventions to support smoking cessation initiatives. The aim of the current study was to to assess the relationship between nicotine dependence and willingness to quit smoking in a sample of adult Saudi population and identify the primary motivations and barriers affecting their decision and willingness to quit smoking.

While it is widely accepted that smoking cessation is an important factor that can be intervened for patients diagnosed with smoking - attributable diseases, reducing the risk of complications and improving treatment outcomes, the current success rate of smoking cessation is quite unsatisfactory. Nicotine dependence is an important risk factor for smoking and the failed attempts to quit smoking among adults who smoke (Raw et al., 1998; Huo et al., 2023). The current study utilized the FTND to assess the level of nicotine dependence among participants. The FTND is a subjective and non-invasive test used for assessing the consistency of physical addiction to nicotine. It provides a systematic measure of nicotine dependence to determine the appropriate methods for clinical intervention (Heatherton et al., 1991; Fagerstrom et al., 2012).

In the current study sample, 13 % of the participants showed high level of nicotine dependence. Participants' demographic characteristics as well as the duration of smoking and level of nicotine dependence did not significantly correlate with the decision and willingness of the participants to quit. In a study conducted by Almogbel et al. (2016) it was reported that, among a sample of 467 male Saudi college students, addiction level was associated with a lower willingness to quit smoking. Potential explanations for the discrepancy between findings of both studies might be the differences in participants' characteristics and sample size.

Concern for personal health was the main reason for the willingness to quit smoking in the current study. This finding aligns with previous reports from other countries and regions (Yang et al., 1996; Ling et al., 2021). This consistent association highlights the importance of health-related factors in smoking cessation efforts. To reduce the health burden of smoking, people who smoke in KSA need to be educated about the long-term risks associated with tobacco use, including cancer, stroke, and cardiovascular diseases. Evidence from the literature underscores the potential benefits of quitting smoking, even after extended periods of use, as cessation significantly lowers the risk of these life-threatening conditions (Lam et al., 2007; Godtfredsen et al., 2008; Jiang et al., 2010). These findings, together with the current study's observations, emphasize the urgency of implementing targeted cessation programs and health communication strategies aimed at promoting smoking cessation by increasing awareness about the positive health outcomes associated with quitting.

The results of the present study also indicated that, key barriers to the willingness to quit smoking were mainly related to social factors such as the enjoyment derived from smoking and friends who smoke. Those social factors are considered drivers that create a reinforcing environment that normalizes and sustains smoking behaviour. Additionally, psychological factors such as depression and fears of failure or weight gain further complicate the motivation to quit since smoking may be used as a coping mechanism in those positions. Previous reports also suggested that depression, in particular, is associated with lower smoking cessation success rates, nevertheless, quitting smoking could attribute to reduced depression, anxiety, and stress, and improved mood and quality of life, with interventions like psychosocial mood management and pharmacological treatments potentially enhancing cessation outcomes for individuals with depression (Taylor et al., 2014; Weinberger et al., 2020). Addressing these interconnected factors is essential to designing effective smoking cessation interventions that are supposed to not only target individual behaviours but also address the broader social and psychological contexts of the smoking habits.

In conclusion, the findings of the present study provide insights into the factors influencing the willingness to quit smoking. While demographic characteristics, duration of smoking, and nicotine dependence level did not significantly correlate with participants' willingness to quit, personal health concerns was a key motivator. Significant barriers to the smoking cessation decisions appeared to be related to social influences and psychological challenges. These interconnected factors highlight the need for comprehensive smoking cessation interventions considering each of these various aspects in order to eventually improve patients' health. Limitations of the current study include the small sample size and reliance on self-reported data which may introduce recall bias, limiting the generalizability and precision of the findings.

## CRediT authorship contribution statement

**Afraa Murriky:** Project administration, Methodology, Investigation, Conceptualization. **Eman Allam:** Writing – review & editing, Writing – original draft, Validation. **Hind Alotaibi:** Methodology, Conceptualization. **Razan Alnamasy:** Methodology, Formal analysis, Conceptualization. **Atheel Alnufiee:** Investigation, Data curation. **Amjad AlAmro:** Writing – original draft, Data curation. **Amal Al-hammadi:** Validation, Investigation.

## Funding

This research did not receive any specific grant from funding agencies in the public, commercial, or not-for-profit sectors*.*

## Declaration of competing interest

The authors declare that they have no known competing financial interests or personal relationships that could have appeared to influence the work reported in this paper.

## Data Availability

Data will be made available on request.

## References

[bb0005] Almogbel Y., Abughosh S., Almeman A., Sansgiry S. (2016). Factors associated with the willingness to quit smoking among a cohort of university students in the KSA. J Taibah Univ Med Sci..

[bb0010] Banks E., Joshy G., Weber M.F. (2015). Tobacco smoking and all-cause mortality in a large Australian cohort study: findings from a mature epidemic with current low smoking prevalence. BMC Med..

[bb0015] Curry S., Wagner E., Grothaus L. (1990). Intrinsic and extrinsic motivation for smoking cessation. J. Consult. Clin. Psychol..

[bb0020] Etter J.F. (2005). A comparison of the content-, construct- and predictive validity of the cigarette dependence scale and the Fagerstrom test for nicotine dependence. Drug Alc Dependence..

[bb0025] Fagerstrom K., Russ C., Yu C.R., Yunis C., Foulds J. (2012). The Fagerstrom test for nicotine dependence as a predictor of smoking abstinence: a pooled analysis of varenicline clinical trial data. Nicotine Tob. Res..

[bb0030] Godtfredsen N.S., Lam T.H., Hansel T.T. (2008). COPD-related morbidity and mortality after smoking cessation: status of the evidence. Eur. Respir. J..

[bb0035] Heatherton T.F., Kozlowski L.T., Frecker R.C., Fagerström K.O. (1991). The Fagerström test for nicotine dependence: a revision of the Fagerström tolerance questionnaire. Br. J. Addict..

[bb0040] Huo X., Li X., Gu M., Qin T., Qiao K., Bai X., Wang Y., Yang Y. (2023). Mechanism of community quitters’ psychological traits on their smoking cessation effects: based on a study of community intervention. Tob. Induc. Dis..

[bb0045] Jiang Y., Elton-Marshall T., Fong G.T., Li Q. (2010). Quitting smoking in China: findings from the ITC China survey. Tob. Control..

[bb0050] Lam T.H., Li Z.B., Ho S.Y. (2007). Smoking, quitting and mortality in an elderly cohort of 56,000 Hong Kong Chinese. Tob. Control..

[bb0055] Li S.J., Wang N.N.Q.X.X. (2020). Research progress in acupuncture treatment of tobacco dependence. Chin J. Tradit. Chin. Med..

[bb0060] Ling L., Haifeng L., Ying Z., Chengyuan X., Houyun U., Zizhen C. (2021). Exploring the degree of nicotine dependence and willingness to quit smoking in Chinese smoking patients with stroke: A cross-sectional survey. Medicine.

[bb0065] Lucchiari C., Masiero M., Pravettoni G. (2020). Psychological and behavioral correlates of readiness to stop smoking. J. Addict. Nurs..

[bb0070] Ma H.Q., Han L., Jin Q.J. (2020). The relationship between nicotine dependence and the psychological characteristics of smokers in the community. Chin J Gen Pract..

[bb0075] Mushtaq N., Beebe L.A. (2017). Psychometric properties of Fagerström test for nicotine dependence for smokeless tobacco users (FTND-ST). Nicotine Tob. Res..

[bb0080] National Center for Chronic Disease Prevention and Health Promotion (2019).

[bb0085] Raw M., McNeill A.N., West R. (1998). Smoking cessation guidelines for health professionals—a guide to effective smoking cessation interventions for the health care system. Thorax.

[bb0090] Siddiqi K., Husain S., Vidyasagaran A. (2020). Global burden of disease due to smokeless tobacco consumption in adults: an updated analysis of data from 127 countries. BMC Med..

[bb0095] Taylor G., McNeill A., Girling A., Farley A., Lindson-Hawley N., Aveyard P. (2014). Change in mental health after smoking cessation: systematic review and meta-analysis. The BMJ..

[bb0100] Weinberger A., Chaiton M., Zhu J., Wall M., Hasin D., R. Goodwin. Trends in the prevalence of current, daily, and nondaily cigarette smoking and quit ratios by depression status in the U.S.: 2005-2017 (2020). Am. J. Prev. Med..

[bb0105] Xu T.M., Zhao J., Chang H. (2018). A qualitative study on influencing factors of smoking cessation behavior in young and middle-aged stroke inpatients. J. Nurs. Sci..

[bb0110] G. Yang, J. Ma, A. Chen. Smoking cessation in China: findings from the 1996 national prevalence survey. Tob. Control. 10(2001), pp. 170–4. 10.1136/tc.10.2.170 PMC1747542 11387539

